# Factors associated with full immunization of children 12–23 months of age in Ethiopia: A multilevel analysis using 2016 Ethiopia Demographic and Health Survey

**DOI:** 10.1371/journal.pone.0225639

**Published:** 2019-11-27

**Authors:** Yohannes Kinfe, Hagazi Gebre, Abate Bekele

**Affiliations:** Department of Biostatistics, School of Public Health, College of Health Sciences, Mekelle University, Mekelle, Tigray, Ethiopia; University of Ghana College of Health Sciences, GHANA

## Abstract

**Background:**

Only 40% of World Health Assembly member states achieved 90% national full vaccination coverage in 2015. In the African region, 79% of the countries had not achieved the target in 2015. In Ethiopia, only 39% of children 12–23 months of age were fully vaccinated. Though different studies were conducted in Ethiopia, they were limited in scope and used single level analysis. Therefore, this study aimed to assess individual and community level factors associated with full immunization among children 12–23 months of age in Ethiopia.

**Methods:**

The data was obtained from Ethiopia Demographic and Health Survey 2016, conducted from January 2016 to June 2016. The sample was taken using two stage stratified sampling. In stage one, 645 Enumeration Areas and in stage two 28 households per Enumeration Area were selected systematically. Weighted sample of 1929 children 12–23 months of age were included in the study. Data was extracted from http://www.DHSprogram.com. Multilevel logistic regression was employed. Akaike Information Criteria was used to select best fit model.

**Results:**

Mother’s education, husband employment, mother’s religion, mother’s antenatal care visit, presence of vaccination document, region and community antenatal care utilization were significantly associated with children full vaccination. The odds of full vaccination were 2.5 [AOR = 2.48 95% CI: 1.35, 4.56] and 1.6 [AOR = 1.58 95% CI: 1.1, 2.28] times higher in children of mothers with secondary or higher and primary education respectively than children of mothers with no education.

**Conclusion:**

This study showed that children full vaccination is affected both by the individual and community level factors. Therefore, efforts to increase children full vaccination status need to target both at individual and community level.

## Introduction

Immunization is a well-established and the most cost-effective way of controlling and eliminating life threatening infectious diseases [1]. It is estimated that it prevents between 2 and 3 million deaths each year [1]. Immunization protects 2–3 million children each year from killer childhood diseases like measles, diarrhea and pneumonia [2]. Investing one dollar on childhood immunization saves 44 United States dollar (USD) which could have been lost due to medical costs and productivity loss [2].

Although tremendous efforts and progresses have been made, about 19.5 million children worldwide still do not receive even the most basic vaccines. These children are vulnerable to dangerous and killer diseases. Currently, 1.5 million children globally die from vaccine preventable diseases each year [2].

The 2016 global vaccine action plan (GVAP) report showed that only 40% of the 194 World Health Assembly member states achieved 90% full vaccination coverage as per their national programs in 2015. This is less than the achievements of 2013 and 2014 which was 43% in both years. Above all, there are disparities between the World Health Organization (WHO) regions and countries. In the African region, 79% of the countries have not achieved the target in 2015 [3,4].

Another global routine vaccination report revealed that, among children worldwide who did not receive the most basic vaccines during their first year of life, 61% live in 10 countries including Ethiopia. The largest proportions were in the WHO African region (17%). These indicated that generally Africa and specifically Ethiopia is lagging behind the global progress towards the achievement of GVAP 2011–2020 target which recommends all countries to reach 90% or more national full vaccination coverage by 2020 [5,6].

Preventable communicable diseases including vaccine preventable diseases of children are still continuing to be the major health problem of Ethiopia [7]. Children not fully vaccinated are vulnerable to killer diseases such as pneumonia and measles which could have been prevented by routine vaccination [8]. According to the 2016 Ethiopia Demographic and Health Survey (EDHS) report, 1 out of 15 Ethiopian children die before reaching age five [9]. This ranges from 39 deaths per 1000 live births in Addis Ababa-the region with highest full vaccination coverage to 125 deaths per 1000 live births in Afar- the region with lowest full vaccination coverage [9].

There are some studies concerning immunization undertaken in Ethiopia in different settings. The determinant factors identified by these studies were, region, access to media, antenatal care (ANC) visit, poverty, place of delivery, mothers education, maternal age and child’s birth order. However, these studies were analyzed using single level analysis which does not consider the hierarchical structure of the data. This can lead to wrong conclusion. In addition to this, most of the studies are limited in scope [10–13].

Therefore, this study was intended to investigate factors associated with childhood full vaccination in Ethiopia both at individual and community level using multilevel analysis of the EDHS 2016 data. The EDHS data has nested structure which makes it best suited for multilevel analysis. Multilevel analysis of individual and community level factors does not depend on the assumption of independence of observations. Therefore, the nature of the EDHS data fits best with multilevel modeling [14].

## Methods and materials

### Data source and study subjects

The data source was the 2016 EDHS. It is the fourth and most recent nationally large scale dataset of demographic and health survey. It was conducted by the Central Statistical Agency (CSA) from January 18, 2016 to June 27, 2016. It has taken nationally representative sample from all the nine regions and two administrative cities of Ethiopia [9].

The sample was taken using a two stage stratified sampling. Each region was stratified into urban and rural areas, which yielded 21 sampling strata. Samples of Enumeration Areas (EAs) were selected in each stratum in two stages. In the first stage, 645 EAs (202 urban and 443 rural) were selected. A household listing operation was implemented in the selected EAs, and the resulting lists of households served as the sampling frame for the selection of households in the second stage [9].

In the second stage, a fixed number of 28 households per EA were selected with an equal probability systematic selection from the newly created household list. All women aged 15–49 years who were usual members of the selected households were eligible for female survey. All men aged 15–59 years who were usual members of the selected households were eligible for male survey. Children of age 12–23 months with missing age of child and outcome variable were excluded from the study. A total weighted sample of 1929 children 12–23 months of age were included in the study [9].

The relevant data (children recode) on vaccination of children 12–23 months of age was extracted from the EDHS 2016. This was done after being registered and sending the concept note of this study through website in order to get permission from Inner City Fund (ICF) international to access and use the dataset. After obtaining permission, the dataset was downloaded from the website at http://www.DHSprogram.com [9].

### Study variables

For this study, review of related literatures and the questionnaire used by the EDHS 2016 were used to select appropriate variables. The Demographic and Health Survey (DHS-7) recode manual was used for appropriate coding of the variables. The outcome variable is child’s full vaccination status. It is a binary outcome variable that is coded as “1” to mean fully vaccinated if the child received at least one dose of Bacille Calmette-Guerin (BCG), three doses of DPT, three doses of polio and one dose of measles vaccine or “0” to mean fully unvaccinated if the child missed one or more doses of the above listed vaccines [9].

Because children full vaccination is not only affected by individual level factors, this study examined the effect of both individual and community level factors affecting children full vaccination simultaneously using multilevel model [15]. The individual level variables are further categorized to parent related and child related characteristics. These individual level variables are: mother’s age, mother’s education, mother’s occupation, husband’s education, husband’s occupation, marital status, religion, ANC visit, wealth index, mother’s empowerment in decisions, media exposure, presence of vaccination document (source of information), child’s birth order, sex of child, place of delivery and perception of distance to a health facility.

The EDHS data have only two community level variables that directly measure the community characteristics. These are the place of residence (rural or urban) and region (either of the nine regions or the two administrative cities). Therefore, we created community level variables by aggregating the individual level’s characteristics with in their respective clusters. These were calculated using the average values of the proportions of individuals in each category of respective variable. In reference to national median values of the proportions, the aggregate values were categorized into high or low. These aggregate community level variables are: community media exposure, community women education, community unemployment proportion, community poverty status, community ANC utilization, community institutional delivery and community perceived distance to health facility.

### Statistical analysis

Analysis was done using STATA version 13. To adjust the non-proportional allocation of the sample to different regions and their rural and urban areas, weighting was applied. So, the representativeness of the survey results both at the national and regional levels is guaranteed. Frequencies and percentages were used to describe the categorical variables. Data was presented using tables.

Historically, multilevel problems were being analyzed by aggregating or disaggregating the measurement values of all variables to one single level of interest which then is followed by an ordinary multiple regression, analysis of variance, or some other ‘standard’ analysis methods. But, bringing and analyzing variables from different levels at one single common level is prone to fallacies of results. Ecological fallacy: This type of fallacy occurs when aggregated data are interpreted at the individual level. Atomistic fallacy: This type of fallacy occurs when inferences are made at a higher level based on analysis performed at a lower level. In addition to this, multilevel data like that of the 2016 EDHS concerns a population with a hierarchical structure. In such samples, the individual observations are not independent and hence violate the assumption, independence of observations which is required by most statistical analysis techniques [15,16].

To overcome the above stated problems faced when dealing with hierarchically structured data such as the 2016 EDHS, using multilevel modeling is more appropriate. This type of modeling enables to precisely estimate the standard errors without the need to stick to the assumption of independence of observations [15]. Therefore, this study applied multilevel binary logistic modeling for the binary response of the outcome variable-child’s full immunization status. A mixed model involving two levels—individuals nested within communities was fitted.

The log odds of children full immunization was modeled using [15]:

Yij=γ00+γp0Xpij+γ0qZqj+u0j

Where: Yij is full immunization status of i^th^ child in the j^th^ cluster whereas γ00 is the intercept; that is the probability of immunization in the absence of explanatory variables. The term γp0 is the regression coefficient of the individual level variable Xp and γ0q is the regression coefficient of the community level variable Zq. Xp and Zq are individual and community level explanatory variables respectively. The term u0j is the community level error and the subscripts i and j represent for the individual level and for the cluster number respectively.

The clustering effect or community variation was estimated using the Intra-class Correlation Coefficient (ICC)–the percentage of variability explained by the upper level (community). ICC was calculated using the formula [15]:

$$ICC=\frac{V_{A}}{V_{A}+\frac{\pi^{2}}{3}}$$

Where: *V_A_* is community level variance and $\frac{\pi^{2}}{3}$ is individual level variance which is equal to 3.29 (the value of $\frac{\pi^{2}}{3}$ in case of standard logistic distribution) [15].

Assuming varying intercept across communities but fixed coefficients, four models were developed. The first one is the Null model; this is a model with no explanatory variables whereas Model I is a model with only individual level variables. Model II is a model constructed with community level variables only and Model III is a combined model which is developed by combining both the individual and community level variables together.

Null model (the model with no explanatory variables) was fitted to estimate the clustering effect or between community variation and to justify the application of multilevel analysis by determining ICC. Accordingly, about 50% of the total variation in the odds of children full vaccination is due to community difference. This high ICC value and significant community variance (P<0.001) justifies that the application of multilevel model is appropriate. The variance which is due to clustering effect decreased from 49.88 in the null model to 23.19, 22.98 and 19.25 in model I, model II and model III respectively (Table 1).

**Table 1:** Random effects estimates of individual and community level factors of children full vaccination in Ethiopia 2016.

Estimates	Null model	Model I	Model II	Model III
Community variance	3.275***	0.993***	0.981***	0.784***
ICC (%)	49.88	23.19	22.98	19.25
PCV (%)	Reference	69.68	70.04	76.06
AIC	2380.59	1863.82	2071.61	1821.26

Key: AIC–Akaike Information Criteria ICC—Intraclass Correlation Coefficient

***–P-value < 0.001 PCV–Percentage Change in Variance

To explore the relative contribution of individual and community level variables in explaining children full immunization, Proportional Change in Variance (PCV) was calculated in reference to the null model. It was calculated as: $PCV=\frac{Vo-Vi}{Vo}$ [15] Where: *Vo* is variance in the null model and *Vi* is variance in the consecutive models. Model III has highest PCV showing that individual and community level variables combined together have higher contribution in explaining the variation in children full immunization than either of them alone (Table 1).

A model with lowest Akaike Information Criteria (AIC) was considered as the best fit model [15]. Therefore, the combined model was taken to be the best fit model. This model showed that more than three-fourth (76.06%) of the total variance in the odds of children full vaccination is attributed to both the individual and community level variables. Even though the unexplained community variance is reduced in the combined model it still remained significant (p<0.001). This implies that there are still uncontrolled community level factors that could explain the variability in the odds of children full vaccination.

The measures of association (fixed-effects) between the odds of children full vaccination and the different explanatory variables were expressed as Adjusted Odds Ratio (AOR) with their 95% CIs and p-value 0.05 was set to be a cut point for statistical significance. The presence of multicollinearity among independent variables was checked using Variance Inflation Factor (VIF) at cut off point of 10. There was no predictor variable with a VIF value of 10 or more indicating the absence of multicollinearity between the predictor variables [17]. Interaction among the explanatory variables was assessed and no significant interaction was found.

Ethical approval was obtained from ethical review committee of Mekelle University, College of Health Sciences with approval and supporting letter. To register and get permission to use the EDHS data, the title and concept note of the thesis proposal was sent through the DHS website. Then permission to use the EDHS data, an authorization letter was obtained from ICF international. The EDHS data has no individual identifiers which could affect confidentiality of participants. Accessed data was used for the purpose of this study only and the EDHS data set was not shared with third person without direct registration.

## Results

### Descriptive results

Only 39% of Ethiopian children 12–23 months age were fully vaccinated.

#### Individual level characteristics of study subjects

The median age of respondents was 28 years and 50% of the respondents were in the age group between 25 and 34 years. About two-third of the respondents participated in this study (62.7%) had no formal education. More than half of the respondents (54.3%) were unemployed in the last twelve months prior to the survey. Nearly half (47.8%) of the mothers’ husbands had no formal education. About one-tenth (9.1%) of the mothers’ husbands were unemployed in the last twelve months prior to the survey. It was found that 94.7% of the mothers were living with a partner. About four in ten (39.2%) of the mothers were Muslims. About one-third (33.2%) of the respondents had attended ANC at least four times during their latest pregnancy. One-fourth (25.1%) of the mothers were from households with poorest wealth index. More than eight out of ten (81.3%) respondents were not exposed to mass media (either radio or television). One-third (33.6%) of the mothers had vaccination document of their children. Nearly one-third (30.1%) of the study children were second or third born children. Above half (53.8%) of the study children were females. About two-third (65.3%) of the children were born at home. Six in ten (59.9%) mothers had perceived distance to health facility as a big problem for them (Table 2).

**Table 2:** Individual level characteristics of children 12–23 months of age in Ethiopia, 2016 (n = 1929).

Individual level variables	Frequency (n)	Percentage (%)
**Maternal age**		
15–19	81	4.2
20–24	400	20.7
25–29	573	29.7
30–34	439	22.8
35–39	284	14.7
40–44	114	5.9
45–49	38	2.0
**Maternal education**		
No education	1210	62.7
Primary	555	28.8
Secondary or higher	164	8.5
**Maternal employment status**		
No	1048	54.3
Yes	881	45.7
**Husband education**		
No education	873	47.8
Primary	718	39.3
Secondary or higher	235	12.9
**Husband employment status**		
No	166	9.1
Yes	1660	90.9
**Marital status of mother**		
Living with partner	1826	94.7
Not living with partner	103	5.3
**Religion of mother**		
Orthodox	671	34.8
Protestant	429	22.2
Muslim	756	39.2
Other	73	3.8
**ANC visit**		
None	685	37.4
1–3 times	540	29.4
> = 4 times	608	33.2
**Wealth index**		
Poorest	485	25.1
Poorer	381	19.8
Middle	433	22.4
Richer	353	18.3
Richest	277	14.4
**Media exposure**		
Not exposed	1569	81.3
Exposed to either media	360	18.7
**Presence of vaccination document**		
No	1281	66.4
Yes	648	33.6
**Birth order number**		
1	358	18.6
2–3	581	30.1
4–5	442	22.9
> = 6	548	28.4
**Sex of child**		
Male	892	46.2
Female	1037	53.8
**Place of delivery**		
Home	1260	65.3
Health facility	669	34.7
**Health facility distance (perception)**		
Big problem	1156	59.9
Not big problem	773	40.1

Key: ANC = Antenatal Care

#### Community level characteristics of study subjects

About nine in ten (88.4%) of the respondents were rural residents. Out of the total respondents, 83.0% were from Amhara, Oromiya and SNNPR regions only. About half (48.8%) of the respondents were from communities with high proportion of women unemployment. Above half (50.8%) of mothers were from communities with high proportion of poverty. More than four in ten (42.8%) of mothers were living in communities having high proportion of ANC utilization (Table 3).

**Table 3:** Community level characteristics of children 12–23 months of age in Ethiopia, 2016 (n = 1929).

Community level variables	Frequency (n)	Percentage (%)
**Type of place of residence**		
Urban	223	11.6
Rural	1706	88.4
**Region**		
Tigray	146	7.6
Afar	19	1.0
Amhara	351	18.2
Oromiya	848	44.0
Somali	73	3.8
Benishangul	20	1.1
SNNPR	403	20.9
Gambela	5	0.2
Harari	5	0.2
Addis Ababa	50	2.6
Dire-Dawa	9	0.4
**Community media exposure**		
Low	1051	54.5
High	878	45.5
**Community women education**		
Low	883	45.8
High	1046	54.2
**Community women un-employment**		
Low	987	51.2
High	942	48.8
**Community poverty**		
Low	948	49.2
High	981	50.8
**Community ANC utilization**		
Low	1104	57.2
High	825	42.8
**Community institutional delivery**		
Low	914	47.4
High	1015	52.6
**Community perceived distance to health facility**		
Low	973	50.4
High	956	49.6

Key: ANC = Antenatal Care SNNPR = Southern Nations Nationalities and Peoples Region

#### Individual and community level factors affecting children full vaccination

As is presented in Table 4 below, educational level of mother, husband employment status, religion of mother, ANC visit and presence of vaccination document from individual level factors whereas region and community ANC utilization from community level factors were statistically significant factors affecting children full vaccination (Table 4).

**Table 4:** Individual and community level factors associated with full vaccination of children 12–23 months of age in Ethiopia, 2016 (n = 1929).

Individual level variables	Vaccination status Frequency (%)		COR [95% CI]	AOR [95%CI]
	No	Yes		
**Maternal education**				
No education	838 (69.2)	372 (30.8)	1	1
Primary	299 (53.9)	256 (46.1)	2.79 [2.08, 3.74]***	1.58[1.1, 2.28]*
Secondary or higher	48 (29.5)	116 (70.5)	7.97 [5.13, 12.39]***	2.48[1.35, 4.56]**
**Husband employment status**				
No	126 (76.1)	40 (23.9)	1	1
Yes	985 (59.3)	675 (40.7)	2.82 [1.8, 4.42]***	2.1[1.32, 3.35]**
**Religion of mother**				
Orthodox	332 (49.5)	339 (50.5)	1	1
Protestant	236 (55.1)	193 (44.9)	0.52 [0.33, 0.83]**	1.03[0.59, 1.78]
Muslim	556 (73.5)	200 (26.5)	0.26 [0.18, 0.38]***	0.8[0.48, 1.32]
Other	61 (84.1)	12 (15.9)	0.09 [0.03, 0.25]***	0.3[0.10, 0.84]*
**ANC visit**				
None	557 (81.3)	128 (18.7)	1	1
1–3 times	320 (59.3)	220 (40.7)	3.96 [2.81, 5.6]***	1.94[1.31, 2.86]**
> = 4 times	248 (40.7)	360 (59.3)	8.16 [5.82, 11.46]***	2.21[1.48, 3.3]***
**Presence of vaccination document**				
No	889 (69.4)	392 (30.6)	1	1
Yes	297 (45.8)	351 (54.2)	3.50 [2.67, 4.59]	1.61[1.21, 2.16]**
**Community level variables**				
**Region**				
Addis Ababa	5 (10.8)	45 (89.2)	1	1
Tigray	48 (32.7)	98 (67.7)	0.18 [0.07, 0.45]	0.93[0.34, 2.51]
Afar	16 (84.8)	3 (15.2)	0.006 [0.002, 0.02]	0.16[0.05, 0.48]**
Amhara	190 (54.2)	161 (45.8)	0.06 [0.02, 0.15]	0.51[0.18, 1.42]
Oromiya	639 (75.3)	209 (24.7)	0.02 [0.009, 0.06]	0.32[0.12, 0.88]*
Somali	57 (78.2)	16 (21.8)	0.01 [0.005, 0.03]	0.33[0.11, 0.97]*
Benishangul	9 (42.6)	11 (57.4)	0.11 [0.04, 0.28]	1.22[0.43, 3.47]
SNNPR	214 (53.1)	189 (46.9)	0.08 [0.03, 0.19]	0.72[0.26, 2.0]
Gambela	3 (58.9)	2 (41.1)	0.03 [0.01, 0.09]	0.3[0.10, 0.85]*
Harari	3 (57.8)	2 (42.2)	0.07 [0.02, 0.19]	0.44[0.16, 1.27]
Dire Dawa	2 (24.2)	7 (75.8)	0.38 [0.14, 1.09]	1.88[0.63, 5.66]
**Community ANC utilization**				
Low	826 (74.9)	277 (25.1)	1	1
High	359 (43.5)	446 (56.5)	8.78 [6.0, 12.83]	1.56[1.06, 2.28]*

Key

* = p<0.05

** = p<0.01

*** = p<0.001

ANC = Antenatal Care AOR = Adjusted Odds Ratio CI = Confidence Interval

COR = Crude Odds Ratio

SNNPR = Southern Nations, Nationalities and Peoples Region

The odds of full vaccination were 2.5 [AOR = 2.48, 95% CI: 1.35, 4.56] and 1.6 [AOR = 1.58, 95% CI: 1.1, 2.28] times higher in children born to mothers who had secondary or higher education and primary education respectively as compared to those born to mothers with no education. Mothers having employed husbands were 2 [AOR = 2.1, 95% CI: 1.32, 3.35] times more likely to fully vaccinate their children than those who have unemployed husbands. Children of Catholic/traditional/other religions following mothers were 70% [AOR = 0.3, 95% CI: 0.1, 0.84] less likely to be fully vaccinated than those of Orthodox followers. The odds of full vaccination were 2.2 [AOR = 2.21, 95% CI: 1.48, 3.3] and 1.9 [AOR = 1.94, 95% CI: 1.31, 2.86] times higher among children of mothers who had ANC visit of four or more times and 1–3 times respectively as compared to those with mothers who have no ANC visit (Table 4).

Mothers who have shown vaccination document of their children were 1.6 [AOR = 1.61, 95% CI: 1.21, 2.16] times more likely to fully vaccinate their children as compared to those who were not able to show their children’s vaccination document. The odds of full vaccination were 84% [AOR = 0.16, 95% CI: 0.05, 0.48], 70% [AOR = 0.3, 95% CI: 0.10, 0.85], 68% [AOR = 0.32, 95% CI: 0.12, 0.88] and 67% [AOR = 0.33, 95% CI: 0.11, 0.97] less in children from Afar, Gambela, Oromiya and Somali regions respectively than those who were from Addis Ababa (Table 4).

Children of mothers from community with high proportion of ANC utilization were 1.6 [AOR = 1.56, 95% CI: 1.06, 2.28] times more likely to be fully vaccinated as compared to children of mothers from community with low proportion of ANC utilization (Table 4).

## Discussion

This study attempted to identify both the individual and community level determinant factors of children full vaccination simultaneously. Accordingly, maternal education, employment status of husband, religion of mother, ANC visit of mother and presence of vaccination document were individual level factors that affect children full vaccination in Ethiopia. Region and community ANC utilization were community level variables that could explain the variation in children full vaccination among communities.

The current study showed that maternal education was significantly associated with children full vaccination. The odds of full vaccination were higher among children of mothers having an educational level of secondary or higher and primary than children of mothers having no formal education. This is in line with studies conducted in Mozamibique [18], Kenya [19], Nigeria [20], Southwest Ethiopia [13], north central Nigeria [21] and Indonesia [22]. This could be due to the reason that more educated mothers are more informed and aware of the advantage of vaccination and its schedule. On the other hand, a study from Uganda found no significant association between mother’s education and children full vaccination [23]. The reason for this might be due to that the participants of the study from Uganda were almost from similar category of educational level. Eight in ten (79.8%) were from incomplete primary education leading them to have similar experiences regarding education [23].

Husband employment status was significantly associated with children full vaccination. The odds of full vaccination were higher in those children of mothers having employed husbands. This might be due to the reason that the husband’s earning eases the transport or other indirect expenses related to vaccination. In addition to this, employed husbands could have better exposure to vaccination related information from their workmates and may become motivated. Whereas a study from Lao PDR found no significant association for husband employment [24]. This difference might possibly be due to the small sample size (317) and single level analysis method of the Lao PDR’s study [24].

Religion was also significantly associated with children full vaccination in Ethiopia. Children born to mothers following Catholic or Traditional religions had lower odds of full vaccination than children born to Orthodox religion following mothers. Other studies conducted in Ghana and Nigeria also found significant association between religion and children full vaccination [25,26].

The current study revealed that children born to mothers who utilized ANC had higher odds of full vaccination than those born to mothers who had not utilized ANC. This is in line with other studies conducted in east China, Indonesia and southwest of Ethiopia [13,22,27]. This might be due to the fact that health professionals provide mothers who come to health facilities for ANC with health education including the advantages and schedule of children vaccination. The other possible reason could be that mothers who visited health facility for ANC could have better experience of visiting health facility and better health seeking behavior.

Presence of vaccination document was significantly associated with children full vaccination. The odds of full vaccination were higher in children born to mothers who had vaccination document as compared to children born to mothers who had no vaccination document in Ethiopia. Study conducted in Ghana and another multilevel study done in Ethiopia using the 2011 EDHS has found similar findings [25,28]. The possible reason for this may be due to that mothers with vaccination document could easily remember their child’s appointment hence help them complete their child’s vaccination. It is easier for the health care providers working in the health facilities to notify and follow the progress of vaccination if children have vaccination document. It might also be easy for the health extension workers to identify and advice mothers with vaccination card at time of house to house visit.

Region of residence was significant predictor of full vaccination in the current study. Children from Afar, Gambela, Oromiya and Somali regions had lower odds of full vaccination as compared to those from Addis Ababa. Similarly, geographical region was identified to be significant predictor of full vaccination in a study conducted in Indonesia and a multilevel study conducted in Ethiopia from the 2011 EDHS [22,28]. The possible reason for these differences could be due to difficulty in accessing health service in the regions with low full vaccination status. Out of 338 rural kebeles in Afar region 24 of them had no health posts. In Afar and Somali regions, where the peoples are predominantly pastoralists, there is seasonal movement of the communities where the Health Extension Workers (HEWs) could not travel with them to provide health services including children vaccination and ANC service [10].

The current study showed that the odds of children full vaccination were higher in those who were from a community with high proportion of ANC utilization as compared to those who were from community with low proportion of ANC utilization. This might be due to the fact that mothers who go to health facility for ANC follow-up become more informed about the benefits and schedule of vaccination. These mothers may also inform other mothers in their area. Therefore, the community becomes informed indirectly.

The results of the current study showed that the community level variance was large and statistically significant. This indicates that there were large differences in children full vaccination among communities which favors the use of multilevel model for this study. It was also indicated by this study that the mixed model (model III) was the best fit model. This indicated that the variation in full vaccination was best explained by the combination of individual and community level factors which supports the use of multilevel model [15,29].

### Limitations

Although the study has got important strengths, it has limitations too. The ICC at the final model was 19.25% and it was significant. This implies that there are still uncontrolled community level factors affecting children full vaccination. Variables perception of provider availability and perception of drugs availability were totally missing. All these variables were excluded from analysis. This study has not assessed supply side factors like type of nearby health facility, type of health professionals providing the vaccination services which could affect full vaccination status. These variables were not included in the EDHS 2016.

## Conclusion

This study found that both the individual and community level factors determine the status of children full vaccination in Ethiopia. Educational level of mother, employment status of husband, religion of mother, ANC utilization of mother and presence of vaccination document at individual level were significantly associated with children full vaccination. Region of residence and community ANC utilization were significantly associated community level factors. Therefore, efforts towards increasing children full vaccination in Ethiopia need to address both the individual and community level factors. The EDHS need to include the supply side factors in the coming surveys. The federal ministry of health needs to strengthen mobile clinics in Afar and Somali regions.

## Supporting information

S1 File Authorization letter. (PDF)

S2 File Child dataset final. (DTA)
